# Caries and white spot lesion trajectories of orthodontic patients across an observation period of 20 years

**DOI:** 10.1007/s00784-024-05752-w

**Published:** 2024-06-11

**Authors:** Carolina Ganss, Nelly Schulz-Weidner, Katharina Klaus, Julia von Bremen, Sabine Ruf, Niko C. Bock

**Affiliations:** 1 Department of Operative Dentistry, Endodontics and Paediatric Dentistry, Section of Cariology, Medical Centre of Dentistry, Philipps University Marburg, Georg-Voigt-Str. 3, 35039 Marburg, Germany; 2 Department for Pediatric Dentistry, Justus Liebig University Giessen, Schlangenzahl 14, 35392 Giessen, Germany; 3 Department of Orthodontics, Justus Liebig University Giessen, Schlangenzahl 14, 35392 Giessen, Germany

**Keywords:** Caries, White spot lesions, Orthodontic treatment, Fixed appliances, Risk indicator

## Abstract

**Objectives:**

Fixed orthodontic appliances may increase the risk for caries and white spot lesions. The aim of this retrospective study was to determine the long-term associations between both in orthodontic patients.

**Materials and methods:**

103 patients aged 36.6 ± 6.5 years whose fixed appliance orthodontic treatment had finished at least 15 years ago were included. Current clinical data and photographs (T3), panoramic x-ray and photographs from before treatment (T0), after debonding (T1) and at 2-year follow-up (T2) were available. Parameters of interest were dentine caries, “Missing/Filled Teeth” (MFT), “White Spot Lesion” (WSL) index and “Periodontal Screening and Recording” index (PSR; T3 only).

**Results:**

At T0, 30.4% had no caries experience decreasing to 25.6%, 22.4% and 6.8% at T1, T2 and T3 resp. The median MFT (95% CI) at T0, T1, T2 and T3 was 2 (1;3), 3 (2;4), 3 (2;4) and 7 (6;9) resp. increasing significantly at each time point (*p* < 0.001 each); 30.1% had WSL at debonding. Patients with caries experience at T0 had a 2.4-fold increased risk of WSL at debonding. Dentine caries, caries experience and WSL at T1 were significantly associated with incident caries at T2, but not at T3. PSR at T3 had a significant association with previous caries incidence and WSL.

**Conclusions:**

Caries experience prior to orthodontic treatment may constitute a risk indicator for WSL, and caries experience and WSL at its end for caries incidence in the near term.

**Clinical Relevance:**

Present caries and WSL may help identifying orthodontic patients with special need for prevention and counselling.

## Introduction

Dental caries is a non-communicable dental disease that occurs due to the metabolic activity of dysbiotic biofilms. The change of a non-pathogenic biofilm to a dysbiotic state is caused by a variety of biological or behavioural factors and is a dynamic process that can be reversed by protective factors [1]. Orthodontic appliances can promote dysbiotic conditions [2] and this means that the period during which fixed appliances are in situ may be accompanied by a change in plaque ecology with a potentially greater pathogenicity compared to the state before. This might reverse after debonding.

If the mineral balance is shifted towards a net mineral loss, clinically detectable changes in the tooth structure may occur. Non-cavitated stages, referred to as initial caries lesions, precede the more advanced conditions associated with dentin involvement. Since such initial caries lesions are accompanied by whitish opaque colour changes, the term “white spot lesions (WSL)” is used synonymously [3].

Orthodontic treatment is often associated with the development of WSL around the brackets, with incidence rates reported between 24 and 73% [4]. Caries at typical risk areas such as occlusal and proximal surfaces and caries in the sense of WSL in the context of orthodontic treatment both have the same aetiology and should therefore share common risk factors. Accordingly, factors such as duration of treatment, insufficient oral hygiene, gingivitis and low fluoride exposure have been linked to an increased incidence of WSL [5–9] and fixed appliance treatment appears to increase the incidence of caries compared to untreated individuals [10, 11]; however, no [12, 13] or opposite associations have also been reported [14].

While it is clear that patients who are scheduled for or undergoing treatment with fixed orthodontic appliances generally should receive comprehensive preventive care [15], it would be helpful to identify those who are at particularly high risk of developing WSL and caries both short- and long-term.

For caries risk assessment in general, different frameworks have been designed that synthesise a variety of biological and behavioural factors to predict future caries, but there is still insufficient evidence on the validity of such tools [16, 17]. Many of these include caries experience, which seems to be one of the most valid predictors of future caries, at least when applied as a single factor [18]. WSL incidence during orthodontic treatment and its possible association with post-orthodontic changes of WSL especially in cases with caries experience has been studied very little. A cross-sectional study with 130 patients was able to provide evidence of a significant association between the number of carious molars at the beginning of orthodontic treatment and the prevalence of WSL after debonding [19]. However, longitudinal and long-term data are missing.

For natural reasons, the primary goal is to prevent the development of WSL during orthodontic treatment from the outset. Newer procedures, such as infiltration [20], can achieve good results in case of WSL occurrence, but they require a relatively aggressive pre-treatment, such as etching with hydrochloric acid, and also incur significant costs for the patient. However, WSL can also regress over time even without further intervention [21, 22]. It would therefore be valuable to have a predictor for such therapy-independent potential improvements in the long-term, not least to avoid overtreatment. To the best of the authors’ knowledge, however, no such studies regarding this question exist up to date.

As part of a previous long-term observational study [23] we found a significant proportion of post-orthodontic WSL to improve without treatment [22], and we also found a significant relationship between the prevalence of WSL and caries [22]. Thus, even though WSL are a much-discussed topic in the context of fixed orthodontic appliances especially in the anterior area, caries in other regions of the dentition should also be given attention.

We have extended data from a previous study [22] and performed a more in-depth analysis of caries and WSL trajectories over an observation period of about 20 years. The aim of our retrospective study was to determine the long-term associations between caries and WSL and associated risk indicators. The 20-year observation period extends from the start of multibracket appliance treatment to debonding, and continues to a shorter follow-up period of 2 years and a further, longer follow-up examination after at least 15 years.

## Participants, materials & methods

**Fig. 1:**
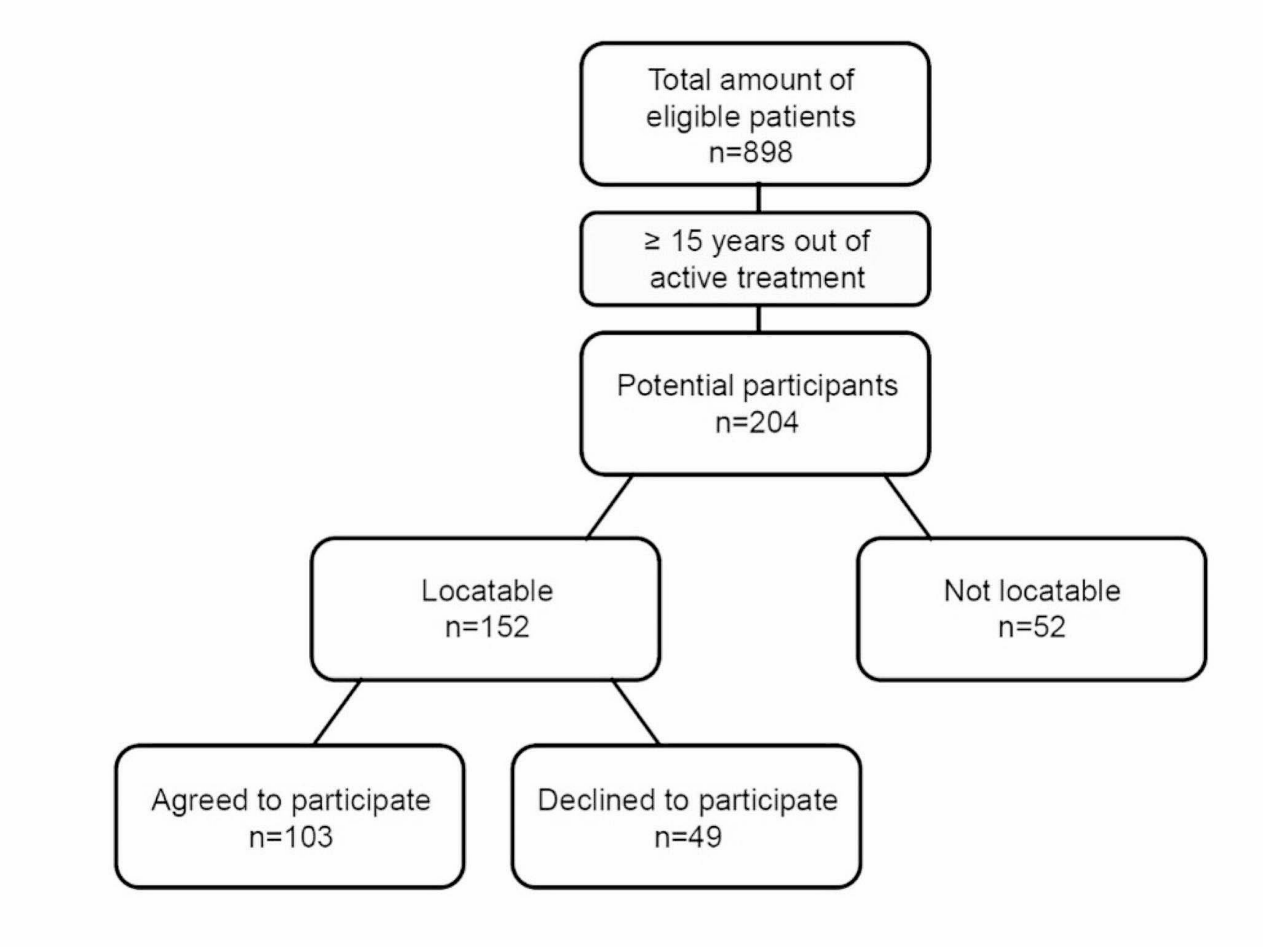
Flow chart for patient recruitment

The present retrospective study utilises panoramic x-rays, photographs and clinical data from two earlier long-term observational studies [22, 23] of orthodontic Class II patients (ethical approval: document number 146/13, Medical Faculty, University of Giessen, Germany; trial registration: DRKS00006354 and DRKS00023022). The general data basis for the current study was generated and published recently [23]. Patients had been invited for a follow-up clinical examination and for intra-oral and extra-oral photographs. Inclusion criteria were patients of any age and sex who had undergone treatment with a Herbst-Multibracket appliance completed at least 15 years ago. All patients gave informed consent to participate. In addition to clinical data, past panoramic x-rays and photographs from routine treatment were retrospectively evaluated. Figure 1 shows the flow chart of patient recruitment. The orthodontic treatment and all investigations were conducted at the Department of Orthodontics, University of Giessen, Germany.

Variables of interest were prevalence and severity of WSL immediately after debonding and at long-term follow-up, caries experience at the beginning of orthodontic treatment, immediately after debonding, at two years after debonding and at long-term follow-up as well as the PSR index [24] at long-term follow-up.

### Assessment of WSL

WSL were assessed on photographs, as described earlier [22]. The photographs included a labial view of the upper and lower anterior teeth and were taken by a qualified photographer in a room with professional photographic equipment under standardised conditions in terms of camera settings and lighting.

**Fig. 2:**
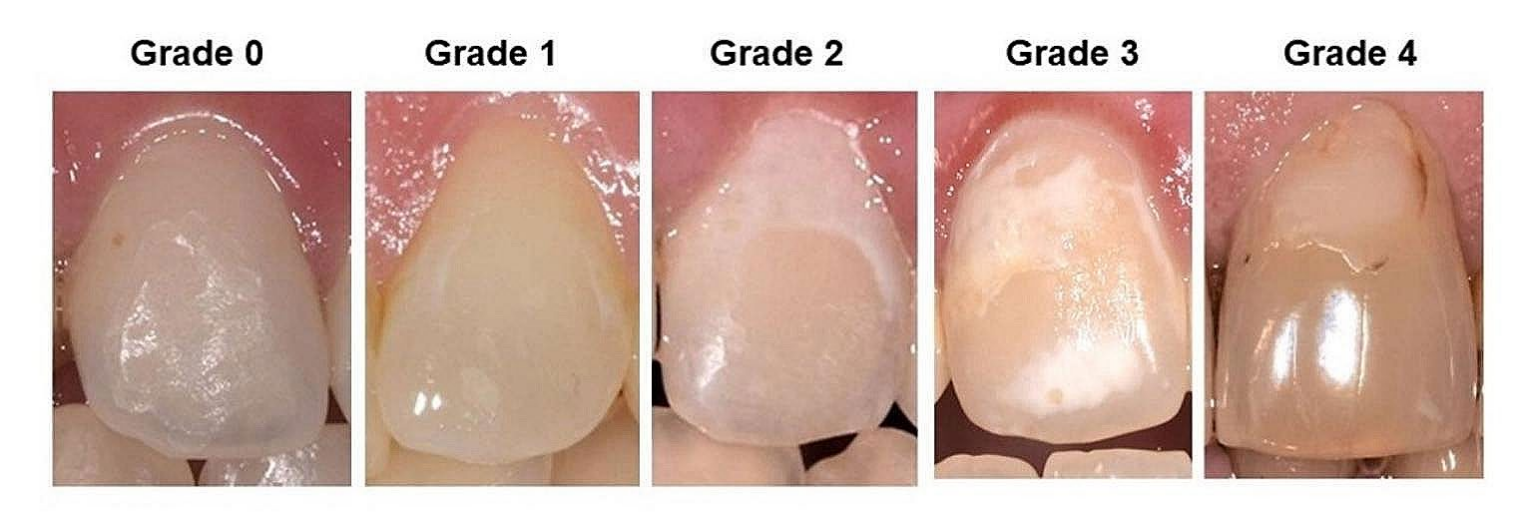
Illustration of the criteria for quantifying WSL; modified version of the WSL-index described by Gorelick: grade 0 = sound, grade 1 = slight whitish alteration, grade 2 = severe whitish alteration, grade 3: whitish alteration with cavitation, grade 4: restorative treatment

The photographs were evaluated under standardised conditions (darkened room, defined distance of the examiners as well as the projection equipment from the screen). The examination was carried out as described earlier [22] by a panel of 5 dentists (J.v.B., K.K., S.R., N.S-W. and C.G.) who had previously been trained in the WSL criteria. For this purpose, two calibration sessions were organised in which photo sets of 6 patients, who did not belong to the group examined here, were evaluated. The results were discussed until mutual agreement was reached. WSL assessment was performed on all incisors at T1 (immediately after debonding) and at long-term follow-up (T3) using a modified version of the WSL index [25] (Fig. 2). WSL were assessed using the index values as well as converting it to a dichotomous variable (WSL at any tooth yes/no). An improvement of WSL was assumed with a difference of the WSL index value T1 - T3 of > 0, this was also used as a dichotomous variable (WSL improved yes/no).

### Assessment of caries experience

Caries experience is defined as the number of teeth/surfaces that had caries lesions, restorations, and/or were missing due to caries, accumulated by an individual [3]. This was assessed on panoramic x-rays before (T0), directly after debonding (T1) and at the follow-up appointment 2 years later. The number of filled (FT) and missing teeth due to caries (MT) was recorded as well as the respective sum (MFT). Caries was assessed at the dentine (D3) level and only as a dichotomous variable (caries present at any tooth yes/no). Incident caries was defined as a carious lesion in dentine on a previously healthy tooth or tooth surface, or a filling on a tooth that had not previously had a restoration.

Panoramic x-rays were assessed by two examiners (N.S.-W., C.G.) independently and blinded to each other’s assessment under standardised conditions (light board, darkened room). Prior to the evaluation, the examination criteria were agreed upon and written down; this protocol was always available when the panoramic x-rays were evaluated. After completion of the assessment, the results were aligned; in cases of disagreement, the panoramic x-rays were re-assessed jointly until agreement was reached. If no agreement could be reached (for example, when deciding whether a tooth was absent or removed because of agenesis, orthodontic indication or other reasons), the patient’s medical record was referred to and an orthodontist (N.C.B.) was consulted.

For T3, only data from the clinical examination were available, which included a dental examination as well as a variety of orthodontic assessments. The examination took place in a dental treatment unit under good lighting and relative dryness with cotton rolls and saliva ejectors. For the present study, the number of filled teeth (FT) and teeth missing due to caries (MT) were used and the MFT index was calculated.

### Periodontal screening and recording (PSR) index

Only data from the clinical examination at the end of the observation period were available for the PSR [24]. The PSR was recorded in four codes (0 = absence of clinical signs; 1 = bleeding on probing; 2 = supra and/or subgingival calculus and/or defective margins; 3 = periodontal pocket 4 mm to 5.5 mm; 4 = periodontal pocket 6 mm or deeper) with a WHO probe and under the conditions described above. Each tooth was probed at the mesiobuccal, midbuccal and distobuccal sites as well as the corresponding oral sites and the highest code of all probing sites was used.

### Statistics

Statistical analysis was performed using MedCalc version 20.251 (MedCalc Software ltd., Ostend, Belgium) and SPSS version 28.0 (IBM, Ehningen, Germany). Since the present study is an explorative retrospective evaluation for which the complete available data were used, no prior sample size calculation was performed.

As caries and WSL data showed a significant deviation from the Gaussian distribution (Kolmogorov-Smirnov-test), non-parametric procedures were used. If not given as frequencies, data are presented as median and 95% confidence intervals obtained by bootstrapping (method of sampling: simple, number of samples: 1000). Age and time periods are given as mean ± SD.

The Wilcoxon-Signed-Rank Tests and Mann-Whitney Tests were used for paired and unpaired comparisons resp. Odds ratios and relative risks were computed for the relationship between the parameters age (< 15 / ≥ 15), gender (female / male), dentine caries (yes / no), caries experience (yes / no) and PSR (PSR 0–1 / PSR > 1) with caries incidence (yes / no) and WSL (yes / no). In addition, Kramer’s Phi was calculated for all cross tabulations. In some cases, panoramic x-rays were partially or completely unavailable (see below). However, as these missing data were completely random, the analyses were carried out with all available data; the respective numbers of cases can be found in the tables. The data on all other variables of interest were complete.

## Results

The group studied consisted of 103 subjects (40.8% males, 59.2% females) who had started orthodontic treatment between 1986 and 2004 (median: 1996). At the beginning of orthodontic treatment, the patients were 15.5 ± 5.9, at the time of debonding 17.9 ± 5.7 and at follow-up 36.6 ± 6.5 years old. The duration of treatment was 28.9 ± 14.7 months and the follow-up period was 18.8 ± 3.2 years resulting in a total observation period of 21.0 ± 3.4 years. Seven of the 103 patients did not have any panoramic x-rays available, but their data were used for the analysis of correlations between WSL and age and gender. For the remaining 96 patients, a panoramic x-ray from T0 was missing in four, that of T1 in six, and that of T2 in 20 subjects. In most patients only one panoramic x-ray was missing, only in four patients two were not available.

**Fig. 3:**
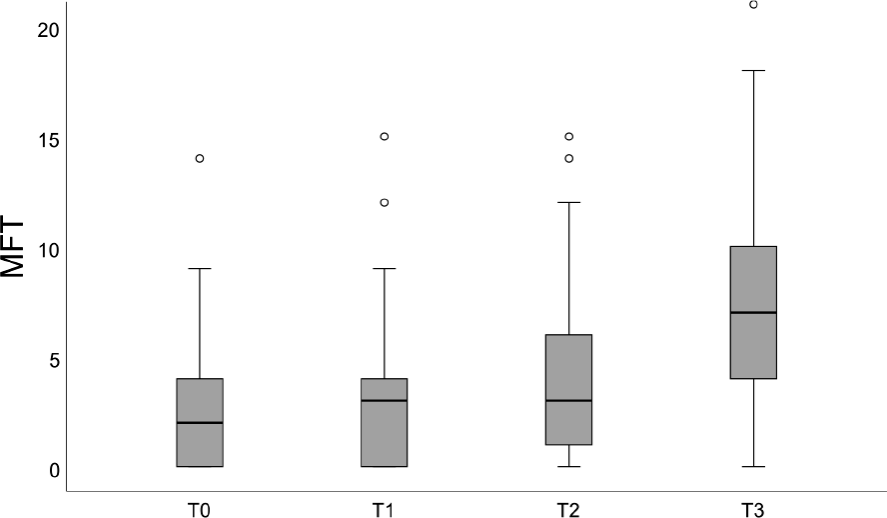
Box- and Whisker-Plots of MFT values (only patients with complete panoramic x-rays; *n* = 69) at the beginning of orthodontic treatment (T0), after debonding (T1), after the first examination period 2 years after debonding (T2) and after long-term follow-up (T3). The box represents the 25th and 75th percentile with median (line), the whiskers minimum and maximum. Circles: outliers

The median MFT (95% CI) at the beginning of orthodontic treatment (T0), after debonding (T1), after the first follow-up 2 years after debonding (T2) and at the end of the observation period (T3) was 2 (1; 3), 3 (2; 4), 3 (2; 4) and 7 (6; 9) respectively (Fig. 3), and increased significantly from one time point to the next (*p* < 0.001 each). Whereas 30.4% of the patients had no caries experience at T0, this value decreased to 25.6% (T1), 22.4% at T2 and further to 6.8% at T3. 30.1% had WSL at debonding.

**Fig. 4:**
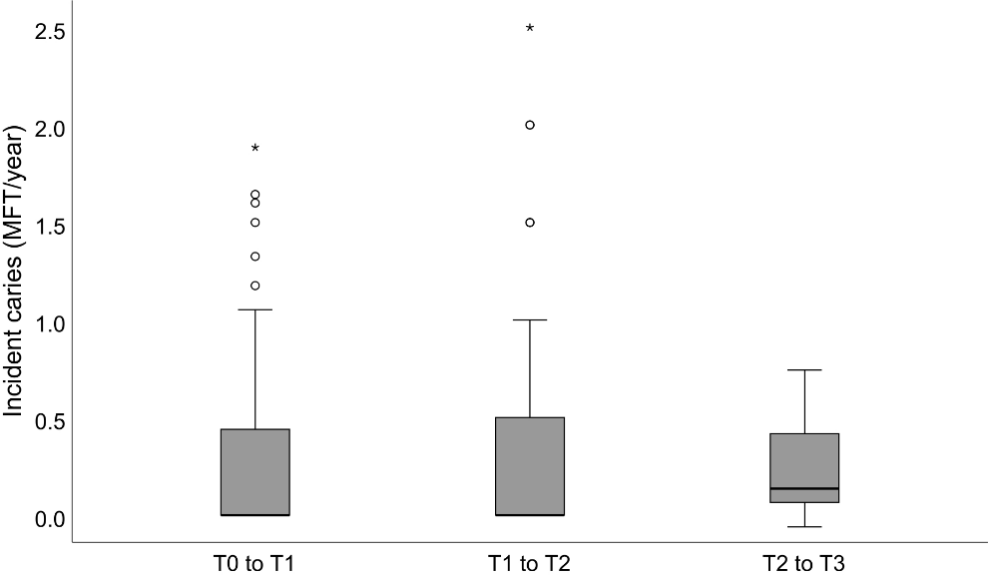
Box- and Whisker-Plots of caries incidence (MFT/year, only patients with complete panoramic x-rays; *n* = 69) during orthodontic treatment (T0 to T1), during the first two years after debonding (T1 to T2), and during long-term follow-up (T2 to T3). The box represents the 25th and 75th percentile with median (line), the whiskers minimum and maximum. Circles: outliers, stars: extreme value

The annual incidence of MFT (Fig. 4) was generally low both during and 2 years after orthodontic treatment (0 (0; 0) MFT each), but showed a wide range. Over the further observation period (T2 to T3), the annual incidence was 0.14 (0.10; 0.18) MFT; no significant differences were found between these time points (T1 to T2: *p* = 0.251; T2 to T3: *p* = 0.710).

### Relationship between baseline parameters and caries/WSL after debonding

Neither dentine caries, caries experience at T0 nor age and gender had a relationship to incident caries at T1 (Table 1). In contrast, there was a significant relationship between caries experience at T0 and WSL at T1. Those who had caries experience at the start of orthodontic treatment were more than twice as likely to have WSL after treatment than those who had not. Furthermore, there was a trend for a somewhat higher risk for younger compared to older patients and for males compared to females, but this missed the significance level by a small margin. There was no association between dentine caries at T0 and WSL at T1 (Table 1).

**Table 1:** Cross tabulations for caries incidence and WSL after debonding (T1) and conditions at baseline (T0); relative risk (RR) and odds ratios (OR) as well as phi values with associated p-values (CE = caries experience)

				RR (95% CI)	OR (95% CI)	Phi (*p* value)
		Incident caries T0 to T1				
		yes	no			
Dentine Caries T0	no (ref.)	16 (24.2%)	50 (75.8%)			
	yes	6 (30.0%)	14 (70.0%)	1.23 (0.56; 2.74)	1.34 (0.44; 4.06)	0.56 *p* = 0.605
CE T0	no (ref.)	5 (18.5%)	22 (81.5%)			
	yes	20 (33.9%)	39 (66.1%)	1.83 (0.77; 4.36)	2.26 (0.74; 6.85)	0.157 *p* = 0.145
Age	≥ 15 (ref.)	14 (28.6%)	35 (71.4%)			
	< 15	11 (29.7%)	26 (70.3%)	1.04 (0.54; 2.02)	0.95 (0.39; 2.57)	-0.013 *p* = 0.907
Sex	female (ref.)	14 (28.6%)	35 (71.4%)			
	male	11 (29.7%)	26 (70.3%)	1.04 (0.54; 2.02)	1.06 (0.41; 2.70)	0.031 *p* = 0.907
		WSL T1				
		yes	no			
Dentine Caries T0	no (ref.)	24 (33.8%)	47 (66.2%)			
	yes	8 (38.1%)	13 (61.9%)	1.13 (0.60; 2.13)	0.83 (0.30; 2.28)	-0.038 *p* = 0.717
CE T0	no (ref.)	5 (17.9%)	23 (82.1%)			
	yes	27 (42.2%)	37 (57.8%)	**2.36** **(1.02; 5.50)**	**3.36** **(1.13; 9.95)**	**0.235** *p* = 0.024
Age	≥ 15 (ref.)	22 (38.6%)	35 (61.4%)			
	< 15	10 (21.7%)	36 (78.3%)	0.56 (0.30; 1.07)	2.27 (0.93; 4.60)	0.181 *p* = 0.066
Sex	female (ref.)	15 (24.6%)	46 (75.4%)			
	male	17 (40.5%)	25 (59.5%)	1.64 (0.93; 2.92)	2.09 (0.89; 4.87)	0.169 *p* = 0.087

### Relationship between caries and WSL after debonding and caries and WSL at further observation times

Those patients with dentine caries at T1 had a more than 4-fold, those with caries experience at T1 an almost 3-fold, and those who had developed WSL a more than 2-fold higher risk of incident caries than those who had neither previous caries nor WSL development. Such relationships disappeared in the further course of the observation (Table 2).

The severity of WSL had improved by the end of the observation period (T3). Of the 32 patients who had WSL after debonding, 12 (37.5%) no longer had detectable WSL at T3. In the remaining 20 (62.5%) who still had WSL, the index score tended to decrease (0.88 (0.5; 1.0) at T1; 0.50 (0.38; 0.75) at T3: *p* = 0.58). The change in WSL, however, was not related to untreated caries or caries experience at T1 (Table 2). Yet, those without improvements had a significantly lower baseline score than those who had improved (0.5 (0.25; 0.63) versus 0.88 (0.50; 1.36); *p* = 0.02).

Even from a retrospective perspective, the PSR index value at T3 had an association with both the caries incidence and with WSL (Table 3). For caries, this was seen in particular during the period from T1 to T2 but also in the overall incidence from T0 to T3. With regard to WSL, patients with a PSR of > 1 had developed almost twice as many WSL at T1 as those with a PSR of ≤ 1.

**Table 2:** Cross tabulations for caries incidence and WSL two years after debonding (T2) as well as at the end of the total observation period (T3) and conditions after debonding (T1); relative risk (RR) and odds ratios (OR) as well as phi values with associated p-values (CE = caries experience)

				RR (95% CI)	OR (95% CI)	Phi (*p* value)
		Incident caries T1 to T2				
		yes	no			
Dentine Caries T1	no (ref.)	3 (5.8%)	49 (94.2%)			
	yes	5 (25.0%)	15 (75.0%)	**4.33** **(1.14; 16.47)**	**5.44** **(1.16; 25.49)**	**0.274** (***p = 0.020)***
CE T1	no (ref.)	3 (15.8%)	16 (84.2%)			
	yes	23 (43.4%)	30 (56.6%)	**2.75** **(0.93; 8.12)**	**4.09** **(1.06; 15.73)**	**0.253** (***p = 0.032)***
WSL T1	no (ref.)	11 (22.4%)	38 (77.6%)			
	yes	15 (65.2%)	8 (34.8%)	**2.91** **(1.59; 5.29)**	**6.48** **(2.18; 19.25)**	**0.415** (***p < 0.001)***
		Incident caries T2 to T3				
		> 2 MFT	0–2 MFT			
CE T1	no (ref.)	6 (31.6%)	13 (68.4%)			
	yes	27 (50.9%)	26 (49.1%)	1.61 (0.79; 3.29)	2.25 (0.74; 6.80)	-0.171 (*p* = 0.146)
WSL T1	no (ref.)	22 (44.9%)	27 (55.1%)			
	yes	15 (55.6%)	12 (44.4%)	1.24 (0.78; 2.00)	0.65 (0.25; 1.68)	-0.102 (*p* = 0.374)
		WSL improved from T1 to T3				
		no	yes			
Dentine Caries T1	no (ref.)	11 (61.1%)	7 (39.9%)			
	yes	7 (70.0%)	3 (30.0%)	1.15 (0.66; 2.0)	1.49 (0.29; 7.74)	0.089 *p* = 0.638
CE T1	no (ref.)	2 (66.7%)	1 (33.3%)			
	yes	16 (64.0%)	9 (36.0%)	0.96 (0.41; 2.25)	1.13 (0.09; 14.2)	0.017 *p* = 0.927

**Table 3:** Relationship of PSR index values at the end of the total observation period (T3) with incident caries (median (95% CI)) and WSL at previous examination dates

	PSR 0–1	PSR > 1	
Incident caries T1	0 (0; 0)	0 (0; 0.5)	*p* = 0.632
Incident caries T2	0 (0; 0)	1 (0; 1)	*p* = 0.001
Incident caries T3	2 (1; 3)	2.5 (2; 5.5)	*p* = 0.268
Incident caries T0 – T3	3 (2; 3)	5 (3.5; 7.5)	*p* = 0.014
WSL T1 no	36 (80.0%)	35 (60.3%)	
WSL T1 yes	9 (20.0%)	23 (39.7%)	phi = -0.211; *p* = 0.033

## Discussion

The study examined a group of orthodontic patients over a period of about 20 years, starting at the age of about 15 and ending at the age of about 36. The aim of the study was to describe the caries and WSL trajectory and to find risk indicators to identify patients with special prevention needs.

The first data of this group of patients date back to the years between 1986 and 2004. This coincides with the beginning of the caries prevention measures implemented by law in Germany in 1988. These include group prevention programmes for 3- to 12-year olds and individual prevention interventions for 6- to 18-year olds. Our patient sample may have already benefited from these measures, but certainly not to the extent that is the case today with full implementation. This is reflected in the relatively wide range of caries experience and in turn makes this group particularly suitable for analyses such as the present one. Population-representative data for 15-year olds are only available for Germany from a cross-sectional study from 2005 (Vierte Deutsche Mundgesundheitsstudie DMS IV [26]), i.e. a decade after the median start of the present study. These data show a higher proportion of individuals without caries experience (64.1% compared to 30.4% in our study) which may be due to the fact that we had panoramic x-rays available, which may have identified more caries than a clinical examination alone, as in the DMS IV. However, the DMS IV also showed a slightly lower MFT (mean 1.5 compared to a mean of 3.2 (median of 2) in our study, respectively). In the same survey, whose sampling time corresponds to the median end of our data sets, 35–44 year olds were also examined. This group had a mean MFT of 14.1 and only 0.7% had no caries experience. The corresponding MFT values at time of follow-up of our sample were only about half of this and the number of subjects without caries experience was considerably higher, which can at least partly be explained by the somewhat younger age of our group. At best, orthodontic treatment at a younger age with frequent treatment appointments and instructions on oral hygiene could also have strengthened the awareness of healthy oral behaviour with the positive effect of a lower caries burden. Overall, however, this comparison shows that our patients represent a group that had a caries prevalence roughly corresponding to the population-representative values.

Our study also included the diagnosis of WSL on photographs. To date, there is no generally established standard method for quantifying WSL, but various approaches all with corresponding advantages and disadvantges have been described [27]. Among other approaches, it has been suggested to quantify the extent of WSL relative to the total crown area. For example, the Enamel Decalcification index [28] divides the tooth surface into four areas and assigns a score for each area (sound, less than 50% of the surface, more than 50% of the surface, decalcification of the total surface or cavity/restoration). Another approach is to use a concentric grid around the centre of the tooth surface [27]. However, we decided to use the Gorelick index [25], partly because of its broad application, but also because of congruence with our previous studies.

No anamnestic data on specific therapies for WSL were available such as infiltration with synthetic materials [20] or the application of peptides [29] for the remineralisation of subsurface structures. Such methods can significantly improve the optical properties of WSL. Although we do not have any information on whether the patients have tried special therapies for their WSL, the systematic review on the effects of infiltration mentioned above [20] only identified clinical studies from 2012 onwards. Since T3 was already completed in 2015 in 24 of the 32 patients with WSL, we can assume that this type of intervention has no relevance for the present study and that we have therefore been able to observe more or less “natural” WSL changes over time.

A retrospective survey, in particular over such a long period of time, always faces the challenge of incomplete patient records, which was also the case in our study. This only concerned the panoramic x-rays, but not the WSL and PSR data. However, since the group as a whole was relatively small and the prevalence of WSL, for example, was quite low, we decided to include also patients with incomplete data whenever possible in order to make the best use of the data set. Furthermore, not all information that would have been helpful was available, such as information on oral hygiene, which could only be deduced from the PSR data collected at the end of the long-term recall. In addition, no clinical findings were available for the caries data survey, only panoramic x-rays. Diagnosing caries experience using panoramic x-rays, however, has advantages and drawbacks. One drawback concerns the survey of fillings potentially resulting in a potential underestimation of filled teeth due to an overlapping with neighbouring teeth or other oral structures and overexposed areas. Furthermore, filling materials differ in terms of their radiopacity. The majority of panoramic x-rays, however, were taken before the year 2000 and thus originated from a time when composites were under development. The composites at that time showed a wide range of radiopacities, mostly with a somewhat higher radiopacity than dentin, but often with one similar to enamel [30]. Thus, composite fillings in posterior teeth may not have been recorded to a certain extent, at least in shallow Class I restorations. However, amalgam was still the standard material for posterior restorations at this time, which was consistent with the findings on the panoramic x-rays. Amalgam has a very high radiopacity [30] compared to enamel and dentin, which made this type of restoration clearly visible in all areas of the panoramic panoramic x-rays. In terms of caries diagnosis, panoramic x-rays have poorer diagnostic accuracy than bitewing x-rays [31], but they can identify non-cavitated dentin caries lesions better than clinical diagnosis alone [32] which is a clear advantage here. However, this would have posed a problem for comparison with the clinical data from T3, so we only used the FT and MT components from these clinical data. As these are very easy to collect, the comparison of radiographic findings and clinical data is likely to be valid.

In general, our data show a continuous caries incidence over the whole observation period. There are not many long-term studies of oral health, for example cohort studies [33], and most cover periods from birth to adolescence but not to later ages. Those with longer follow-up periods beyond the age of twenty have either looked at specific ethnicities (Australia ABS), do not report long-term data for dental caries (Avon Longitudinal Study of Parents and Children (ALSPAC), Iowa Facial Growth Study, Northern Finland Birth Cohort (NFBC), Pelotas Birth Cohort Study (2004)) or report no or only self-reported caries data (British Cohort Study, National Child Development Study (NCDS), Newcastle Thousand Families Cohort Study, Pelotas Birth Cohort Study (1993)).

A large longitudinal study, however, the Dunedin Multidisciplinary Health and Development Study [34], has also assessed caries progression between the age of 15 and 32 years, as we did, along with other age points. In this study, different trajectories were identified where 15.1% of the participants were assigned to a high (DMFS = 42.3 ± 12.7), 44.7% to a medium (DMFS = 18.6 ± 6.8) and 40.2% to a low trajectory group (DMFS = 5.4 ± 3.7). The authors concluded that their study disproved the notion that caries is more a phenomenon of childhood and adolescence, which we can derive in a similar way from our results. A deeper analysis of the data from the Dunedin study [35] showed that, in addition to other factors such as self-assessment of oral health, caries experience in the primary dentition was associated with increased caries experience into middle age. This would support caries experience as a valid parameter for caries risk assessment as mentioned before [18].

We were able to confirm this to some extent as there was no association between caries experience at the beginning and debonding, but with WSL. Further, there were clear associations between caries status as well as WSL at debonding and incident caries at T2. It could be assumed that the fixed bands in the posterior region have a protective effect on the proximal surfaces, while the brackets in the anterior region promote the development of WSL. This would mean that the typical caries risk sites could change during fixed orthodontic therapy. Corresponding differences in caries distribution at the end of orthodontic therapy were shown in a study conducted earlier [36] and the authors hypothesised such effects. After removal of the appliances, the typical caries risk sites may have reappeared, which explains the observations in the period after debonding up to the 2-year follow-up. Thus, patients who have incident of caries and WSL at the time of fixed appliance removal could be identified as caries risk patients who need more intensive preventive care at least in the next few years. However, we were not able to show such relationships for the further observation years from T2 to T3.

With regard to WSL, patients who already had caries experience at the beginning of orthodontic treatment were more than twice as likely to develop WSL than those without caries experience, which identifies these individuals as a WSL risk group with a special need for care, but also with an increased need for counselling. However, how these lesions develop after debonding does not seem to depend on caries status. Overall, more than one third of the patients with WSL no longer had detectable lesions at the end of the observation period, and the severity of the remaining lesions had decreased. However, this was not associated with caries status, but with lesion severity at T1.

WSL are characterised by the disruption of the crystallite structure [37] resulting in the typical whitish-opaque appearance. WSL can be arrested by appropriate caries preventive measures, but the natural structure of healthy enamel cannot be restored. Therefore, WSL remain visible without further intervention like infiltration procedures even when they are arrested. WSL can therefore only disappear or decrease over time due to wear processes such as abrasion [38]. This may also explain our finding that the improvement of WSL is more likely to occur in lesions at higher severity levels that are associated with lower microhardness than those with lower severity. If wear is taken as the main reason for the improvement in WSL, this also explains why caries experience does not play a role here.

An important aspect in our study would have been the oral hygiene status over time, but no concrete/standardized data were available in between the start of treatment and the follow-up two years after debonding. The only information beyond start of treatment was the PSR index obtained as part of the clinical examination at the long-term follow-up. Nevertheless, these data may provide an indirect measure of oral hygiene levels [39] and perhaps of oral health efforts in general. Against this background, a particularly interesting result was that the PSR index at T3 even retrospectively reveals correlations with caries incidence and with WSL. This can only mean that oral hygiene behaviour is a more or less constant factor over longer periods of life, and that a current oral hygiene status also provides information about oral hygiene levels in the past. In the Dunedin study, the plaque level (Simplified Oral Hygiene index- OHI-S) was also examined [40], and three trajectories (low, medium, high) were identified similar to caries. At the age of 32 years, retrospectively to the age of 15 years, the high trajectory group consistently showed mean OHI-S values between 1.3 and 1.7, the medium trajectory group values between 0.8 and 1.2, and the low trajectory group values between 0.4 and 0.8. Increased plaque levels were also associated with a significantly increased incidence risk ratio for caries. Such relationships could also be derived from our data and this would mean that much more effort to improve oral hygiene would be important at a younger age, even if patients have little oral health problems at that time, in order to avoid caries and periodontitis later in life.

A limitation of our study is the relatively small number of cases, which could leave some associations undetected and possibly limit the generalisability of the results in particular to patient groups with either a significantly lower or significantly higher caries prevalence. Furthermore, the patient data were not entirely complete, and the caries parameters collected were relatively crude, with no information available on, for example, oral hygiene and other behavioural and patient-related parameters. The results of our study must therefore be interpreted with appropriate caution. However, they point to clear tendencies that make further studies on this or related questions seem interesting.

## Conclusion

Patients with caries experience at the beginning of orthodontic treatment had a higher risk of developing WSL than those without caries experience. Caries and WSL after debonding could be a risk indicator for caries at least in the short-term. However, this correlation was no longer evident at the long-term follow-up. Since even retrospectively there were still long-term correlations between PSR and incident caries as well as WSL, it seems that the individual oral hygiene level appears to be a permanent condition. This suggests, that more efforts to improve oral hygiene are needed starting from a young age.

## Data Availability

No datasets were generated or analysed during the current study.
